# P-42. Systematic review and meta-analysis of recombinant herpes zoster vaccine in immunocompromised populations

**DOI:** 10.1093/ofid/ofae631.249

**Published:** 2025-01-29

**Authors:** Fawziah Marra, Michael Yip, Jacquelyn Cragg, Nirma K Vadlamudi

**Affiliations:** University of British Columbia, Vancouver, British Columbia, Canada, Vancouver, BC, Canada; University of British Columbia, Vancouver, British Columbia, Canada; University of British Columbia, Vancouver, British Columbia, Canada; University of British Columbia, Vancouver, British Columbia, Canada, Vancouver, BC, Canada

## Abstract

**Background:**

Herpes zoster infection is common in immunocompromised individuals. Recently, the Advisory Committee on Immunization Practices recommended immunizing with the recombinant zoster vaccine (RZV).

**Methods:**

**Objective:** To evaluate the efficacy, immunogenicity and safety of RZV in immunocompromised individuals, such as transplant recipients, cancer patients undergoing chemotherapy, individuals with preexisting autoimmune diseases and HIV-infected patients.

**Data Sources and Study Selection:** From January 1984 to October 2023, a systematic search of PubMed, MEDLINE, EMBASE, Scopus, Web of Science, CINAHL, and Cochrane CENTRAL was performed. Randomized clinical trials (RCT) evaluating RZV compared to placebo in immunocompromised adults were selected.

**Data Extraction and Synthesis:** Study characteristics and estimates on the incidence of herpes zoster, humoral and cellular immune responses, and safety data were extracted from studies. Estimates were pooled using random-effects meta-analysis. Differences by study-level characteristics were estimated using subgroup meta-analysis and metaregression.

**Results:**

Seven RCTs were included. Compared to placebo, RZV reduced the incidence of herpes zoster across all ages by 81% (RR: 0.19, 95%CI: 0.09, 0.44), with moderate heterogeneity across the studies (I2 = 60.49%; τ2 = 0.31; P=0.07). RZV significantly increased humoral and cellular immunity one month after the last dose. Transplant and past malignancy were associated with lower immunogenicity. RZV was more reactogenic with more local and systemic adverse events. There was no difference in serious adverse events or death between the vaccine and placebo arms.

**Conclusion:**

This study suggests that RZV reduces the risk of herpes zoster infection in immunocompromised individuals. This vaccine should be routinely offered to immunocompromised individuals, preferably before chemotherapy or treatment.

**Disclosures:**

**All Authors**: No reported disclosures

